# Effects of polypropylene, polyvinyl chloride, polyethylene terephthalate, polyurethane, high-density polyethylene, and polystyrene microplastic on *Nelumbo nucifera* (Lotus) in water and sediment

**DOI:** 10.1007/s11356-021-17033-0

**Published:** 2021-10-20

**Authors:** Maranda Esterhuizen, Young Jun Kim

**Affiliations:** 1 Aquatic Ecotoxicology in an Urban Environment, Ecosystems and Environment Research Programme, Faculty of Biological and Environmental Sciences, University of Helsinki, Lahti, Finland; 2 Helsinki Institute of Sustainability Science (HELSUS), Fabianinkatu 33, 00014 Helsinki, Finland; 3 Joint Laboratory of Applied Ecotoxicology, Korea Institute of Science and Technology Europe (KIST), Campus 7.1, 66123 Saarbrücken, Germany

**Keywords:** Microplastics, Oxidative stress, Sediment, Macrophyte, Exposure, Germination, Seedling growth

## Abstract

Plastic waste is recognised as hazardous, with the risk increasing as the polymers break down in nature to secondary microplastics or even nanoplastics. The number of studies reporting on the prevalence of microplastic in every perceivable niche and bioavailable to biota is dramatically increasing. Knowledge of the ecotoxicology of microplastic is advancing as well; however, information regarding plants, specifically aquatic macrophytes, is still lacking. The present study aimed to gain more information on the ecotoxicological effects of six different polymer types as 4 mm microplastic on the morphology (germination and growth) and the physiology (catalase and glutathione S-transferase activity) of the rooted aquatic macrophyte, *Nelumbo nucifera*. The role of sediment was also considered by conducting all exposure both in a sediment-containing and sediment-free exposure system. Polyvinyl chloride and polyurethane exposures caused the highest inhibition of germination and growth compared to the control. However, the presence of sediment significantly decreased the adverse effects. Catalase activity was increased with exposure to polyvinyl chloride, polyurethane, and polystyrene, both in the presence and absence of sediment but more so in the sediment-free system. Glutathione S-transferase activity was significantly increased with exposure to polypropylene, polyvinyl chloride, and polyethylene terephthalate in the sediment-free system and exposure to polyethylene terephthalate and polyurethane in the absence of sediment. There was no clear correlation between the morphological and physiological effects observed. Further studies are required to understand the underlying toxicity mechanism of microplastics.

**Supplementary Information:**

The online version contains supplementary material available at 10.1007/s11356-021-17033-0.

## Introduction

The demand for plastics, as versatile polymers with multiple applications, has significantly increased over the years, with polypropylene (PP), polyethylene (PE), polystyrene (PS), polyvinylchloride (PVC), and polyethylene terephthalate (PET) being the major thermoplastics dominating the market (Lithner et al. 2011). In 2019, the global production of plastics reached 370 million tonnes (PlasticsEurope 2019). However, with the one-time-use attitude towards plastic items in conjunction with the current levels of plastic production and low recovery rate, hazardous plastic waste discarded into the environment is sure to increase (Dahlbo et al. 2018; van Velzen et al. 2019), posing a risk to the biota due to entanglement, suffocation or internal damage when consumed (Rochman et al. 2013; Naidoo et al. 2020).

Once discarded in nature, surface embrittled plastics are micro-cracked by climate conditions combined with microbial action resulting in their progressive degradation into the small fragments and particles known as microplastic (MP, particles smaller than 5 mm in size (Arthur et al. 2009)). The degradation may involve hydrolysis of ester bonds, photo-oxidation due to UV exposure, thermal degradation due to heat, and microbial degradation (da Costa et al. 2016; Luo et al. 2018; Ng et al. 2018). During manufacturing, some toxic compounds remain unpolymerised within the plastics. As the plastics degrade, these residual monomers are released into the environment (Revel et al. 2018). Chemicals reported to leach from plastics include bisphenol A, benzene, phthalates, and phenol (Wright and Kelly 2017), and further leaching of toxicants is facilitated by ester bonds hydrolysis (Lithner et al. 2011).

MPs are being detected in nearly all ecosystems across the globe (Barnes et al. 2009; Lusher et al. 2015; da Costa et al. 2016; Imhof et al. 2017; Ng et al. 2018; Peeken et al. 2018; Scopetani et al. 2019), including the water column and sediments of many aquatic environments (Browne et al. 2010; Claessens et al. 2011; Naidoo et al. 2015; Reisser et al. 2015; Hoffman and Hittinger 2017). There is a consensus among the scientific community that even though plastic pollution in aquatic ecosystems is recognised as an environmental threat, data on the ecotoxicity of plastics are scarce (Eerkes-Medrano et al. 2015). Setälä et al. (2014) reported that MP could enter the food chain via ingestion and transferred from one trophic level to the next. Various severely toxic effects have been attributed to MP exposure as well as their leachates in different organisms tested (Pflugmacher et al. 2020a, 2021a, b), but not all (Scopetani et al. 2020a). The presence of MPs also has been reported to disrupt the residence of natural biota and thus could potentially influence biodiversity (Pflugmacher et al. 2020b).

With the detection of MP in the water column (Reisser et al. 2015) and sediments (Scopetani et al. 2019) of aquatic environments, macrophytes are likely to be affected. The majority of the literature on the ecotoxicological impacts of MP has utilised animal systems, and reports on the effects in plants, especially aquatic macrophytes, despite their importance as primary producers in aquatic ecosystems, are limited (Yokota et al. 2017; van Weert et al. 2019). The present study aimed to gain more information on the ecotoxicological effects of different MP polymer types on the sediment-rooted aquatic macrophyte, *Nelumbo nucifera* (Lotus).

*N. nucifera* is India and Vietnam’s national flower and plays a culturally significant role for China and Korea. Lotus flowers are commonly seen in water gardens globally as well as growing wildly in various freshwater habitats. Besides their aesthetic appeal, Lotus plants serve an important role in ecosystems by cooling water temperature and increasing the oxygen status due to leaf cover of the water surface. The plants also provide shelter for fish, protecting against avian predation (Kanabkaew and Puetpaiboon 2004). The potential of using Lotus in wastewater treatment has also been explored (Kanabkaew and Puetpaiboon 2004), especially for removing heavy metals and combatting eutrophication (Mishra 2009; Liu et al. 2013). Lotus plants have been cultured for over 2000 years for their substantial role as sustenance (Guo 2009; Escaray et al. 2012). The Lotus rhizome is consumed as a vegetable and is a source of flour. In many Asian countries and Korea, Lotus root tea or Lotus flower tea is consumed daily (Yu et al. 2002). Lotus seeds are edible and can be consumed fresh or processed into cakes, noodles, fermented rice wine, ice cream, and popcorn. The plants are also important as traditional medicines (La-ongsri et al. 2009), as fresh Lotus seed wine has thirst-quenching, spleen healing, and anti-diarrheal properties (Wu et al. 2013). For the countries that cultivate Lotus on a large scale, the plants are an important export commodity. During cultivation, plastic sheets supported by bamboo arches are used to cover the plants, which has been found to reduce the harvesting time and increase the yield, and plastic sheets are added under the sediments to avoid water and fertiliser losses (Guo et al. 2019). These plastic sheets are likely to degrade to MP under UV irradiation from the sun with time, which could sediment to the rhizomes, especially after biofouling (Fazey and Ryan 2016; Kooi et al. 2017).

The effects of the six most commonly utilised plastics, PP, PVC, polyurethane (PUR), PET, high-density polyethylene (HDPE), and expanded polystyrene (EPS), were assessed as MP (4-mm diameter particles) on the morphology (germination and growth) and physiology (antioxidative enzyme activities of catalase and glutathione S-transferase) of *N. nucifera*. Several factors have been shown to influence the toxicity of MP, including the concentration, shape, environmental ageing and climate of the location where it is discarded (Ziajahromi et al. 2017; Qiao et al. 2019; Pflugmacher et al., 2020a, 2021a, b). However, the focus of this study was to investigate the toxic effects of possible leached chemicals from various polymer types, thus selecting a larger particle size and avoiding media turbulence to assure that any adverse effects observed were not due to uptake. The study did not intend to identify specific mechanisms of toxicity but to compare the toxicities of the six types of MP on one aquatic macrophyte species in terms of germination, growth, and antioxidative enzymes.

## Materials and methods

### Experimental materials

PP MP was derived from the lids of TicTac boxes. Cable isolation was used to produce soft PVC MP, and yellow sponges were used as a source of PUR. From mineral water bottles and their caps, PET and HDPE MP, respectively, were produced. PS was obtained from expanded polystyrene (EPS) packaging. All materials were purchased from the local supermarket, cleaned and washed before shredding to MP on a desktop plastic recycler (SHR3D IT, 3devo B.V. Utrecht, Netherlands) with a sieve size of 4 mm. Smaller particles were removed by manual sieving. Care was taken at all times to avoid self-contamination (Scopetani et al. 2020b). The MP particle size of 4 mm was selected to exclude seed pore blockage (Bosker et al. 2019) or uptake by the sprouting seedlings as the cause for the observed adverse effects, but still small enough to be considered as MP (< 5 mm). All the particles from the various MP types were irregular fragments (Figure S1) formed by shredding. As the shape of MP is known to contribute to the toxicity (Ziajahromi et al. 2017; Qiao et al. 2019), the contribution of this parameter could also be excluded, as all particles were produced in the same way resulting in the formation of the same shape.

Lotus seeds were purchased from BaiLing-Seeds (Yunhe Bailingguangao Sales Department, China). The Lotus seeds were professionally opened by the supplier on one side to allow faster germination. Before the experiments’ inception, the seeds were washed in water and imbibed for 24 h. The lake sediment was collected from Lake Vesijärvi (Lahti, Finland) in 2018. Before the experimental setup, lake sediment was washed by flooding with sterile deionized water, shaking for a day, settling overnight, and decanting. The process was repeated three times. The water from the third washing was microscopically verified not to contain any MP particles. Sediment from an actual lake was used to keep the conditions as close as possible to those in nature.

### Experimental setup

The six treatment groups (one for each MP type) consisted of five replicates, each encompassing a beaker containing 1 g of the various types of MP, respectively, mixed into 5 g of lake sediment plus another 2 g of lake sediment on top (to avoid the MP washing out of the sediment) and 250 mL of standard medium (pH 6.8). The standard medium consisted of 900 mg/L KNO_3_, 900 mg/L Ca(NO_3_)_2_, 360 mg/L MgSO_4_, 200 mg/L KH_2_PO_4_, 40 mg/L Fe-EDTA and micronutrients: 1 mg/L MnSO_4_, 0.2 mg/L CuSO_4_, 0.2 mg/L ZnSO_4_, 1.8 mg/L H_3_BO_3_, 3.4 mg/L (NH4)_6_Mo_7_O_24_, and 9 mg/L CoCl_2_.

For the controls, MP was not added to the 7 g of sediment. A single previously imbibed Lotus seed was embedded in the soil in replicates of five per treatment group. The seeds were incubated at 24 °C ± 0.5 °C and a light–dark cycle of 14:10 h (1500 lx) for 7 days before assessing the germination, seedling growth, and catalase and glutathione S-transferase enzyme activity. The exposure period of 7 days was selected based on the typical germination time of four to five days for Lotus seeds, adding two additional days to allow sprouting of retarded seeds, but not longer to avoid seedlings in the controls outgrowing their exposure vessels.

While a sediment-containing system would more closely represent a realistic environmental scenario, Pflugmacher et al. (2020a) previously demonstrated that the phytotoxicity of MP is buffered in a substrate-containing system and proposed that the leached chemicals likely bind to soil or sediment. To investigate phytotoxicity of the various MP particles without the effect of a substrate, a second exposure was conducted exactly like the first; however, without sediment, i.e., 1 g of MP was suspended in the standard medium and allowed to settle without stirring over the exposure period.

### Morphological effects: germination and growth

After 7 days, the seedlings were carefully removed from the sediment, and the roots were washed with water. The number of germinated seedlings was counted, and the length of each seedling was manually measured in centimetre (cm) with a digital calliper.

Germinated seeds were defined operationally as having a radicle emergence length of 1 mm. The final germination percentage (GP) was determined after 7 days, according to the following formula:

$$GP=\frac{Ngatday7}{Nt}x100$$

where Ng is the number of germinated seeds, and Nt represents the total number of seeds used in the respective batch. The unit for GP is a percentage (%) (Janssen 1973; Scott et al. 1984).

### Physiological effects: catalase and glutathione S-transferase activity

Enzyme extracts were prepared according to Stüven and Pflugmacher (2007) with minor amendments. Seedlings from each treatment individually were ground to a fine powder in liquid nitrogen and suspended in 0.1 M sodium phosphate buffer (pH 6.5) containing 14 mM dithioerythritol and 5 mM EDTA. The solution was homogenised with a glass potter and stirred on ice for 30 min before centrifugation at 5000 × g for 5 min (4 °C). The soluble proteins were precipitated by ammonium sulfate (80% saturation). The soluble proteins were collected in the pellet of a second centrifugation step (25,000 × g, 30 min, 4 °C). The pellet was dissolved in 20 mM sodium phosphate buffer (pH 7.0) and desalted on NAP-10 columns (Amersham Pharmacia, Uppsala, Sweden) before enzyme measurement.

Each replicate’s protein content was determined (Bradford 1976) using the Bradford protein dye reagent (Sigma). Bovine serum albumin (98%, Sigma) was used as a standard for the protein calibration of the assay method. A spectrophotometric assay was performed for the assessments of Catalase (CAT, EC 1.11.1.6) presented by Aebi (1984) and expressed in SI units as µkat/mg protein. The GST (EC 2.5.1.18) assay followed the conjugation rate of 1-chloro-2,4-dinitrobenzene (CDNB) with GSH at 340 nm (extinction coefficient Ɛ = 9.6 L/mmol/cm) according to Habig et al. (1974).

### Statistical data treatment

The data’s homogeneity and normality were evaluated using IBM® SPSS® Statistics Version 25 (2018). Data were tested for normality and homogeneity. Based on the outcomes of these tests, the data were evaluated with the non-parametric Kruskal–Wallis test, followed by a pairwise comparison, to identify statistical significances between the treatment groups and controls. The α-value considered for significance was 0.05 after Bonferroni correction (Sokal and Rohlf 1997).

## Results and discussion

Lotus (*N. nucifera*) plants, native to tropical Asia, grow in waterbodies not deeper than 1 m, with a single flower and leaves on an erect peduncle protruding from a tuberous rhizome that grows in sediment (Imsabai et al. 2013). These Lotus plants are at substantial risk of MP exposure in nature as MP has been detected in sediment from freshwater ecosystems (Klein et al. 2015; Scopetani et al. 2019).

### Morphological effects

**Fig. 1:**
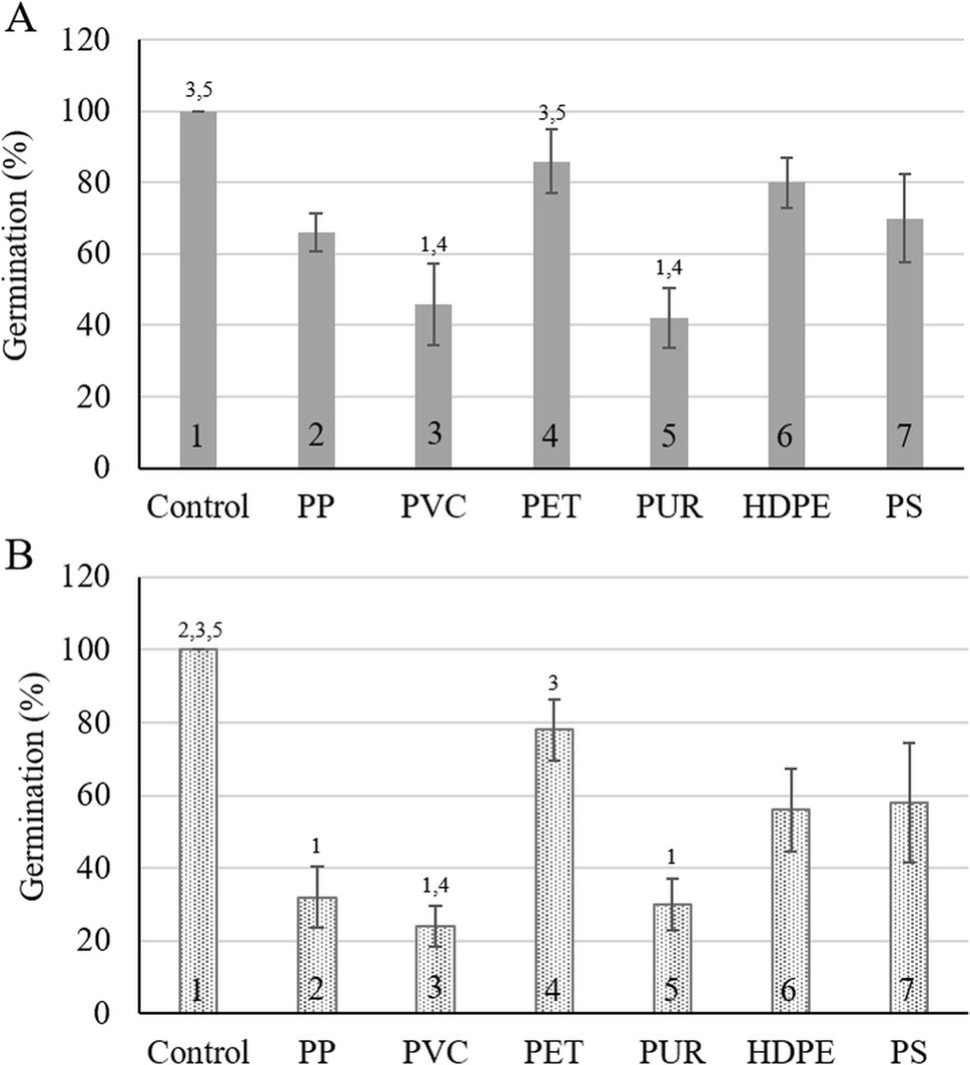
Germination percentage of *Nelumbo nucifera* seeds after 7 days of exposure to six types of microplastic particles in **A** sediment and **B** a sediment-free system. Bars present average germination percentage ± standard deviation (*n* = 5). Numbers above the bars indicate statistical significance (*p* ≤ 0.05) compared to 1: control, 2: polypropylene, 3: polyvinyl chloride, 4: Polyethylene terephthalate, 5: polyurethane, 6: high-density polyethylene, 7: polystyrene

**Fig. 2:**
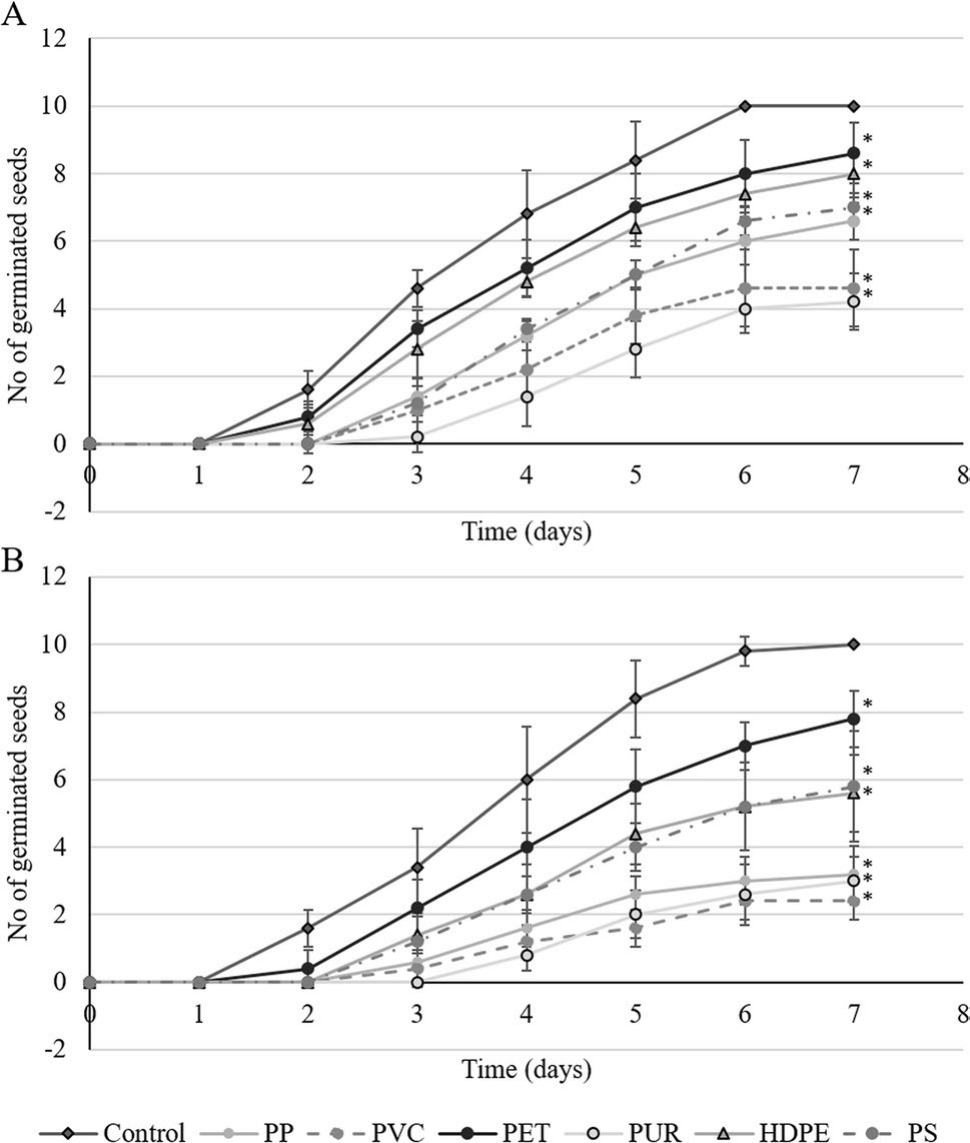
Number of germinated seeds over a period of 7 days with exposure to seven types of microplastic against an unexposed control in **A** sediment, and **B** a sediment-free system. Data points present the average number of germinated seeds ± standard deviation (*n* = 5). Statistical significance (*p* ≤ 0.05) compared to the control is presented by an asterisk (*)

**Fig. 3:**
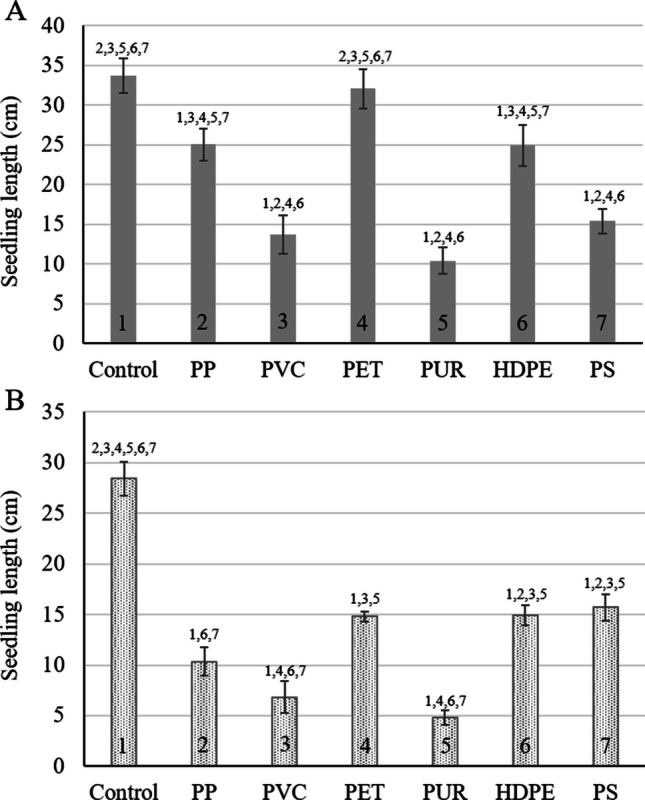
The length of the *Nelumbo nucifera* seedlings after 7 days of exposure to six types of microplastic particles in **A** sediment, and **B** a sediment-free system. Bars present average seedling length ± standard deviation (*n* = 5). Numbers above the bars indicate statistical significance (*p* ≤ 0.05) compared to 1: control, 2: polypropylene, 3: polyvinyl chloride, 4: Polyethylene terephthalate, 5: polyurethane, 6: high-density polyethylene, 7: polystyrene

The effects of the six MP types on the germination percentage of *N. nucifera* were tested both in the presence (Fig. 1A) and absence (Fig. 1B) of sediment. In both control groups, irrespective of the presence or absence of sediment, 100% of the seeds sprouted after 7 days. The overall pattern of the extent to which each MP type affected germination remained the same when comparing the two systems, both for germination (Figs. 1 and 2) and seedling growth (Fig. 3).

The presence of MP in soil was proposed to change soil properties, limit nutrient availability, and affect the native microbiota (Rillig et al. 2019), which would negatively impact plant growth. Considering the influence of the various MP polymer types used in the present study, the role of the sediment as a buffer was evident as significantly more severe germination inhibition in the absence of sediment was observed after 7 days (*p* < 0.001; Fig. 1). For example, PP caused no inhibitory effects in the presence of sediment (*p* = 0.154), but in the absence of sediment, the PP resulted in a 68% reduction in the germination percentage of the lotus seeds (*p* = 0.008). With PVC exposure, inhibition resulted both in the presence (54%; *p* = 0.001) and absence (76%; *p* < 0.001) of sediment, with the presence of sediment dampening the adverse effects on germination. In sediment, PUR caused 58% inhibition (*p* < 0.001), whereas the inhibition was increased to 70% (*p* = 0.004) in the absence of sediment. However, PET (*p* = 1), HDPE (*p* = 1), and PS (*p* = 0.390 for sediment; *p* = 1 for sediment free system) exposures did not influence the germination percentage of the Lotus seeds in either system. Pflugmacher et al. (2020a) investigated the effect of PC leachate and MP granules on the germination of *Lepidium sativum* and similarly found that in a substrate-containing system, the toxic effect of the plastic was reduced. The study by Pflugmacher et al. (2020a) also illustrated that leachates of PC were more toxic than the particles, attributing the toxic effects of the MP to leaching chemicals rather than the physical particles.

When ranking the various plastic types based on their inhibition of the germination percentage in the presence of sediment, the highest-ranked MP would be PUR, eliciting the most severe effects, followed by PVC, with PP, PS, HDPE, and PET eliciting no statistically significant adverse effects. In the absence of sediment, the following changes to the ranking occurred starting with PVC, PUR, followed by PP, but HDPE, PS, and PET exposures did not hinder germination.

Figure 2 illustrates that all the MP polymer types tested significantly retarded the germination of the *N. nucifera* seeds compared to the control (p < 0.05), and in the system without sediment (Fig. 2B), the seeds exhibited a noticeable delay in and slowed germination compared to the sediment containing system (Fig. 2A).

After exposing pre-imbibed Lotus seeds to MP in the presence and absence of sediment, all emerging seedlings in all treatments were alive, and no severe chlorosis or other defects were observed except for reduced growth. Apart from the reduced growth, no visible differences were observed, such as defects to the root and stalks or discolouration. As with the germination percentage findings, seedling length followed a similar pattern for both the effects of the plastic types and the presence and absence of sediment. The absence of sediment amplified the inhibition effects of the various plastic types compared to the exposure system containing sediment. In the sediment-containing exposure system (Fig. 3A), the ranking of the MP types causing the most growth inhibition to the least (or no effect) were PUR (69.2% inhibition, *p* < 0.001), PVC (59.4%, *p* < 0.001), PS (54.4%, *p* < 0.001), HDPE (26.1%, *p* < 0.0.001), PP (25.8%, *p* = 0.001), and lastly PET, which caused no inhibition (*p* = 1).

In the absence of sediment (Fig. 3B), the inhibition ranking was as follows, with the highest inhibition calculated for PUR (83.0%, *p* < 0.001), followed by PVC (76.2%, *p* < 0.001), PP (63.6%, *p* < 0.001), PET (47.9%, *p* < 0.001), HDPE (47.4%, *p* < 0.001), and the lowest for PS (44.7%, *p* < 0.001).

Zimmermann et al. (2019) tested the baseline toxicity, induction of oxidative stress, and endocrine activity of extracts from various plastic polymers types in vitro. In agreement with the present study’s findings, Zimmermann et al. (2019) reported that extracts from PVC and PUR were the most toxic, whereas PET and HDPE caused little or no toxicity. They reported that the toxicities of low-density polyethylene (LDPE), PS, and PP varied.

In the present study, MPs were administered at a concentration of 142 g/kg (w/w) in the sediment-containing system (14% sediment dry weight) or 4 g/L in the sediment-free system. These concentrations did not prove lethal to the Lotus seedlings as they continued to grow, albeit at a reduced rate. Van Weert et al. (2019) tested the effect of PS MP (20–500 μm, up to 10% dry weight) and nanoplastic (50–190 nm, up to 3% sediment dry weight) on the growth of two sediment-rooted aquatic macrophytes, *Myriophyllum spicatum* and *Elodea* sp. for 21 days. Of the two macrophytes, only *M. spicatum* was affected via reduced shoot length as a function of increasing MP concentration. In the present study with Lotus, the exposure concentration of 14% (w/w) PS MP caused a significant reduction in the seedlings’ length. Kalčíková et al. (2017) examined the effects of PE microbeads on the free-floating macrophyte *Lemna minor*. The microbeads did not affect growth or photosynthesis but resulted in root growth inhibition and damage to the root cells. The germination of the Lotus seeds in this study was not affected by HDPE, but the overall seedling length was reduced in both the sediment-containing and sediment-free treatments. The fresh weight of *Vallisneria natans* seedlings was slightly but still significantly decreased with exposure to PVC MP (1% w/w) (Wang et al. 2021). Likely, plants exhibit different sensitivities towards the chemicals leaching from the MP and would thus be differently impacted in an aquatic environment. Irrespective, a disturbance at the primary producer trophic level would be devastating for the entire aquatic foodweb, illustrating the need to understand the effects of MP on plants.

In the present study, the focus was not on the role of the physical properties of the MP particles, in other words, the size and shape, in the comparison of the toxicity of the various polymer types as these parameters were constant in all treatments. However, the size and shape of the particles do come into consideration when comparing the present study to previous findings. For example, in several studies, MP had no effect on the growth rate of duckweed species, i.e. *Lemna minor* exposed to PE microbeads (Kalčíková et al. 2017; Mateos-Cárdenas et al. 2019) and *Spirodela polyrhiza* exposed to PS spheres (Dovidat et al. 2020). However, hard MPs with sharp, jagged edges can affect root growth by causing physical damage at a cellular level (Kalčíková et al. 2017). As the plastics used in this study were irregular fragments contained in the sediment layer or settled on the bottom of the exposure vessel, the plastic hardness and sharp edges may have damaged the developing roots of the seedlings and contributed to the differences in growth and germination observed per plastic-type exposure. In future studies, microscopic visualization would be needed for confirmation.

### Physiological effects

**Fig. 4:**
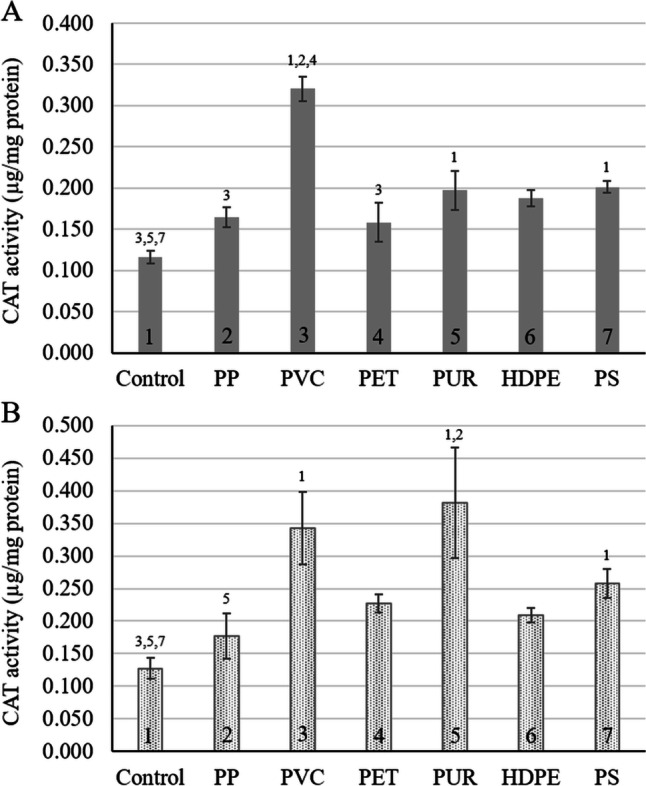
*Nelumbo nucifera* seedlings’ catalase activity after 7 days of exposure to six types of microplastic particles in **A** sediment, and **B** a sediment-free system. Bars present average CAT activity ± standard deviation (*n* = 5). Numbers above the bars indicate statistical significance (*p* ≤ 0.05) of each treatment compared to 1: control, 2: polypropylene, 3: polyvinyl chloride, 4: Polyethylene terephthalate, 5: polyurethane, 6: high-density polyethylene, 7: polystyrene

The physiological effects, measured as the effects on two antioxidative enzymes’ activities, differed significantly from the morphological effects. In both the presence (Fig. 4A) and absence of sediment (Fig. 4B), only exposure to PVC (sediment *p* < 0.001, sediment-free *p* = 0.037), PUR (*p* = 0.050, *p* = 0.001), and PS (*p* = 0.013, *p* = 0.021) caused a significant increase in the CAT activity. PVC exposure caused the CAT activity in the Lotus seedlings to increase by 63%, both in the presence and absence of sediment. Exposure to PUR resulted in a 41% increase in the CAT activity in seedlings cultivated in sediment and a 66% increase in those cultivated in the sediment-free system. PS elevated the catalase activity of the seedlings in sediment by 42% and those in the sediment-free system by 50%.

**Fig. 5:**
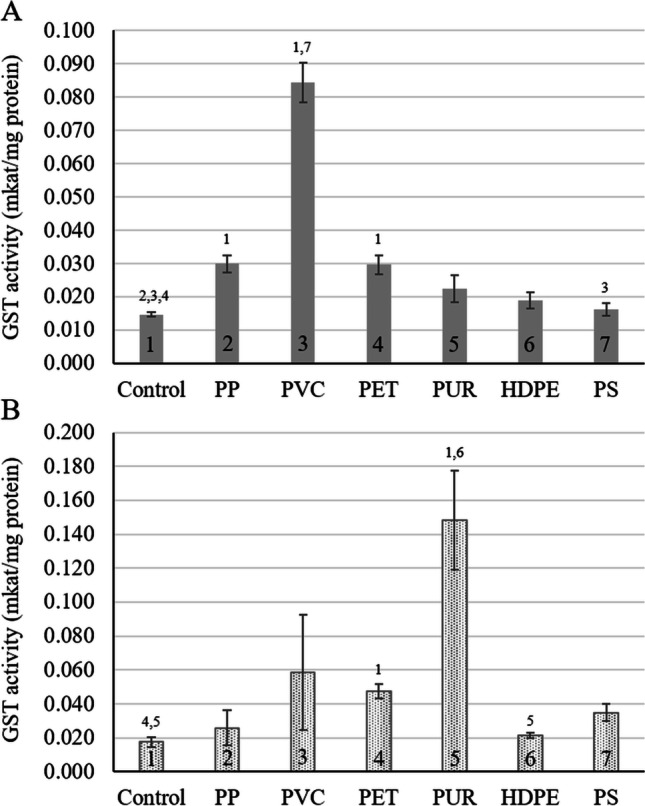
Glutathione S-transferase activity in *Nelumbo nucifera* seedlings after 7 days of exposure to six types of microplastic particles in **A** sediment, and **B** a sediment-free system. Bars present average GST activity ± standard deviation (*n* = 5). Numbers above the bars indicate statistical significance (*p* ≤ 0.05) compared to 1: control, 2: polypropylene, 3: polyvinyl chloride, 4: Polyethylene terephthalate, 5: polyurethane, 6: high-density polyethylene, 7: polystyrene

The GST activity of the seedling in sediment significantly increased by 50.8% with PP (*p* = 0.021), by 82.6% with PVC (*p* < 0.001), and by 50.3% with PET (*p* = 0.044) exposure (Fig. 5A). However, for the exposure system lacking sediment, GST activities were only elevated in seedlings exposed to PET (*p* = 0.020) and PUR (*p* < 0.001) by 63.3% and 88.2%, respectively. Again, the seedlings in the sediment-free system showed a higher GST activity with MP exposure than the respective controls (Fig. 5B).

Wang et al. (2021) reported no increase in the activities of the oxidative stress enzymes superoxide dismutase (SOD) and peroxidase (POD) when exposing *Vallisneria natans* to 5 g PVC MP per 500 g sediment (1%) for 14 days. In the present study, PVC exposure elicited increased CAT and GST activities in Lotus seedlings; however, in the present study, the enzyme activities were measured after 7 days of exposure. To the best of our knowledge, no other studies reported on the oxidative stress status of macrophytes exposed to MP. However, Jiang et al. (2019) studied the effects of various sizes and concentrations of PS on *Vicia faba* L or better known as the fava bean. With exposure to 5 µm PS MP, the CAT activity in the roots of *V. faba* decreased, whereas the SOD and POD activities increased. Exposure to PS nanoplastics promoted the antioxidative enzyme activities of *Cucumis sativus* L. and *Oryza sativa* L. (Li et al. 2020; Zhou et al. 2021).

Oxidative stress plays a role in the toxicity of MP, but the exact mechanism is not fully understood (Hu and Palić 2020). MP causes reactive oxygen species (ROS) generation and accumulation, inducing the activities of the antioxidative defence enzymes (Jeong et al. 2016, 2017). However, whether oxidative stress is a consequence of MP toxicity or causes the ecotoxicity observed is unclear. Increased antioxidative enzyme activities are typically inferred as an indication of oxidative stress (e.g. Rillig et al. 2019), and lipid and protein oxidation likely could contribute to inhibited germination and seedling growth. Conversely, increased antioxidative enzyme activities may indicate oxidative signalling that does not necessarily imply damage to cellular components, and hence oxidative stress (Foyer and Noctor 2005). In the present study, no correlation could be drawn between the inhibitions on the seed germination and growth and the activities of the two enzymes tested. For example, the dominant growth and germination inhibition compared to the control were observed with PUR and PVC exposure; however, PUR and PVC did not significantly affect the GST activity in the sediment-containing or sediment-free exposures. Furthermore, PS exposure did not lead to significant growth or germination inhibition but resulted in a significant increase in CAT activity irrespective of the presence or absence of sediment. Also, PET exposure had no significant morphological adverse effects but elicited the GST activity compared to the control.

### General discussion on ecotoxicological risk assessment

Plastics are considered biochemically inert (Hammer et al. 2012); however, in the environment, polymers are likely to degrade via biotic and abiotic processes (Lambert et al. 2014), releasing hazardous additives (Lithner et al. 2011). Nonetheless, after 7 days of exposure, degradation is unlikely. Sub-micron sized or nanoparticle additives, which are not chemically bound to the polymer, are used in commercial thermoplastic applications more frequently (Sherman 2012). As the additives are typically lipophilic, they can be transferred to fat in a relatively short time (Bieber et al. 1985). These compounds can penetrate cell membranes and interfere in biochemical reactions inducing toxic effects (Hammer et al. 2012). However, Fang (2012) commented that the internalisation of nanoparticles would be limited in plants due to protection provided by cell walls as a physical barrier.

In the study by van Weert et al. (2019), significant effects on the growth of an *Elodea* sp. were not evident from PS MP exposure, even with the highest exposure concentration of 10%. The presented study aimed to compare the toxicities of various MP types; therefore, a higher concentration was selected to observe the differences in toxicities. Scopetani et al. (2019) reported an average MP concentration of 396 MP particles/kg in sediment collected from Lake Vesijärvi (Lahti, Finland). Using the conversion presented by Besseling et al. (2019), i. e. using a weight of 5 µg/particle, the concentration reported by Scopetani et al. (2019) amounts to 2 mg/kg. Klein et al. (2015) reported MP concentrations of up to 1 g/kg in sediment collected on the shore of the Rhine (Rhine-Main Area, Germany). In the freshwater environment, up to 2561 MP particles/m^3^ have been reported in lake water samples in Asia and Europe (Cera et al. 2020). Using the Besseling et al. (2019) conversion, this amounts to 12.8 µg/L. In the current study, a concentration of 142 g/kg (14%) was used in sediment and 4 g/L in water. Therefore, even with the predicted future increase in environmental MP in mind, the results should not be used to evaluate ecotoxicological risk but to compare the effects elicited by the plastic types.

Considering the ecosystem services of Lotus in the aquatic environment of regulating the water temperature, oxygen status, and role in habitat structure and providing shelter, the morphological effects of the MP on germination and seedlings become significant. Reduced germination and plant growth brought about by MP exposure could thus negatively impact the hydrology of MP contamination aquatic ecosystems as well as biodiversity. With Lotus contributing to the economy as an export commodity and food source, reduced yields due to MP exposure could also have secondary adverse effects.

## Conclusion

The presented study aimed to fill in the knowledge gap regarding the toxicity of various polymer types of MPs on plants, specifically aquatic macrophytes. PUR and PVC caused the most significant inhibition of seed germination and seedling growth with no apparent correlation to the trends seen in the elicitation of antioxidative stress enzymes. The mechanistic toxicity of MP needs to be tested against more macrophytes, as based on the current knowledge of studies investigating the effects of MP on macrophytes, some effects could be species-specific. The exact mechanism of inhibition is not yet understood; however, based on the MP size used in the present study, the effects seen are unlikely due to uptake of the MP particles, and 7 days of exposure is expectedly too short a period to induce leaching. However, nanoparticle additives not chemically bound to the polymer or unpolymerised residual monomers within the plastics are likely to be involved. The decreased germination and growth also may be attributed to physical damage at a cellular level brought about by the jagged edges of the irregular MP fragments of more rigid, harder plastics; however, this requires future investigation to be justified.

## Supplementary Information

Below is the link to the electronic supplementary material.

Supplementary file1 (DOCX 902 KB)

## Data Availability

The datasets used and/or analysed during the current study are available from the corresponding author on reasonable request.
