# Supplementary material for: Effects of polypropylene, polyvinyl chloride, polyethylene terephthalate, polyurethane, high-density polyethylene, and polystyrene microplastic on Nelumbo nucifera (Lotus) in water and sediment
Source: Environ Sci Pollut Res Int. 2021 Oct 20;29(12):17580–90. doi: 10.1007/s11356-021-17033-0 (PMC8873133; doi:10.1007/s11356-021-17033-0)
Supplement: Supplementary file 1 — Supplementary file1 (DOCX 902 KB) [file 11356_2021_17033_MOESM1_ESM.docx]

Supplementary data


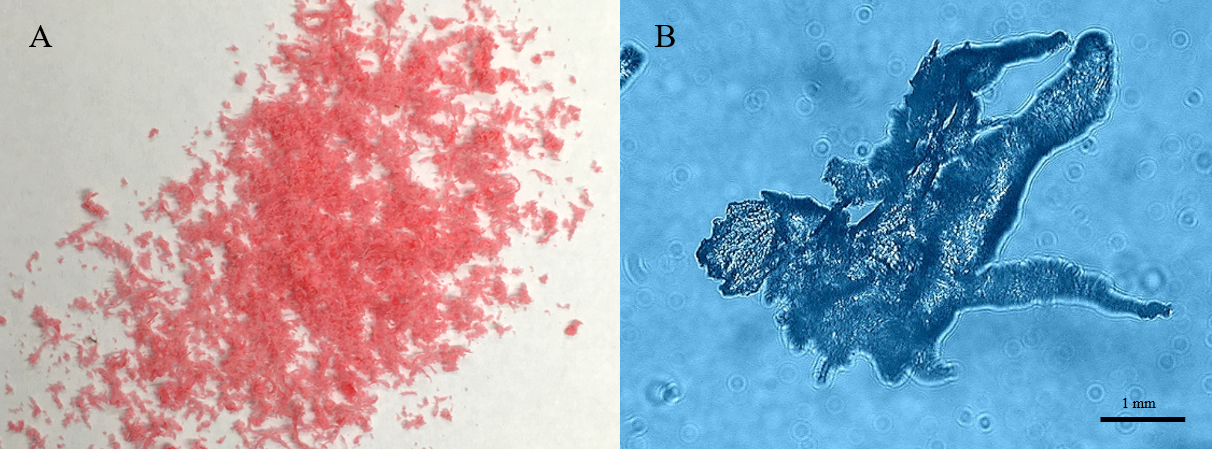


Figure S1: Photograph of the shredded HDPE 4 mm fragments (A) and a microscope image (10x magnification, bright field) of one of these HDPE particles to show the shape of an irregular fragment.
